# Alterations in SiRNA and MiRNA Expression Profiles Detected by Deep Sequencing of Transgenic Rice with SiRNA-Mediated Viral Resistance

**DOI:** 10.1371/journal.pone.0116175

**Published:** 2015-01-05

**Authors:** Cheng Guo, Li Li, Xifeng Wang, Chun Liang

**Affiliations:** 1 Department of Biology, Miami University, Oxford, Ohio, United States of America; 2 State Key Laboratory for Biology of Plant Diseases and Insect Pests, Institute of Plant Protection, Chinese Academy of Agricultural Sciences, Beijing, China; 3 Department of Computer Science and Software Engineering, Miami University, Oxford, Ohio, United States of America; Universidade Federal do Rio Grande do Sul, BRAZIL

## Abstract

RNA-mediated gene silencing has been demonstrated to serve as a defensive mechanism against viral pathogens by plants. It is known that specifically expressed endogenous siRNAs and miRNAs are involved in the self-defense process during viral infection. However, research has been rarely devoted to the endogenous siRNA and miRNA expression changes under viral infection if the resistance has already been genetically engineered in plants. Aiming to gain a deeper understanding of the RNA-mediated gene silencing defense process in plants, the expression profiles of siRNAs and miRNAs before and after viral infection in both wild type and transgenic anti-*Rice stripe virus* (RSV) rice plants were examined by small RNA high-throughput sequencing. Our research confirms that the newly generated siRNAs, which are derived from the engineered inverted repeat construct, is the major contributor of the viral resistance in rice. Further analysis suggests the accuracy of siRNA biogenesis might be affected when siRNAs machinery is excessively used in the transgenic plants. In addition, the expression levels of many known miRNAs are dramatically changed due to RSV infection on both wild type and transgenic rice plants, indicating potential function of those miRNAs involved in plant-virus interacting process.

## Introduction

microRNAs (miRNAs) and small interfering RNAs (siRNAs) are two major classes of small RNAs (sRNAs) that play substantial roles in regulating gene expression transcriptionally and post-transcriptionally [1, 2]. They are often discussed and compared together in many studies because of sharing many common features. For instance, they are both small non-coding RNAs that target mRNAs by recognizing and binding their complementary sequences [3–5]. However, their distinct modes of biogenesis define them as two different classes of sRNAs. Specifically, miRNA is primarily generated from a single-stranded precursor that forms a self-complementary hairpin structure, while siRNA is generated from a double-stranded RNA precursor [6]. In plants, most miRNAs are processed by the Dicer Like Enzyme, specifically DCL1, whereas siRNAs are excised by DCL1 and its homologous proteins (DCL2, DCL3 and DCL4) [7]. After excision, mature miRNAs or siRNAs are loaded onto other protein factors, including the Argonaute proteins, to assemble the RNA-induced silencing complex (RISC) [8]. RISC then leads miRNA/siRNA to pair with specific mRNA targets to execute the translational repression or silencing [9, 10].

RNA-mediated gene silencing is known to serve as a self-defensive mechanism against viral pathogens by host cells. Individuals of *Caenorhabditis elegans* or *Drosophila melanogaster* with mutation affecting RNAi machinery have been reported to be more susceptible to viral infections [11, 12]. Further studies have revealed that such self-defense was because viral RNAs were specifically targeted and silenced by viral induced small interfering RNAs (vsiRNAs) generated in host cells as a defense response to viral infection, which ultimately disturbed the virus replication [13–16]. Thus the mutated individuals became more susceptible to the infection once their RNAi machineries were affected. The biogenesis of vsiRNAs is similar to the aforementioned normal siRNA biogenesis, except the fact that vsiRNA is using an exogenous virus-derived single strand RNA (ssRNA), instead of host genomic sequences, as the template for generation [17, 18]. Besides siRNAs, specific miRNAs have been also reported to possess antiviral capability [16, 19, 20]. During viral infection, numerous known miRNAs species were found to present differential expression profiles that would further influences mRNA expression profiles for defensive purpose [21–25]. Some studies even suggested that novel miRNAs species are induced in host plants during viral infection or extreme stressors, although the actual functions of those novel miRNAs species are not clear yet [26–28]. However, the naturally occurred vsiRNAs- and miRNAs-induced resistance is not enough for protecting the host plants from viral infection.A more effective way is to artificially generate transgenic organisms with viral resistance. One of the common strategies is to integrate an intron-containing hairpin-RNA (ihpRNA) construct into the host plant genome. The ihpRNA construct normally includes a native intron sequence from the host plant genome, which is flanked by two terminal virus-derived sequence fragments that are complimentary to each other to form a hairpin stem structure, hence stimulating specific siRNAs production in host plants for defensive purpose [29, 30]. With a resistant efficiency of ~ 90% to 100% for transgenic plants in most cases, the ihpRNA strategy has been widely used for a variety of crops to combat the virus pathogens [29].

The usage of ihpRNA-based transgenic plants raises an interesting and important biological question: how does the plant natural defensive system react to virus infection when the viral resistance has been already transgenically introduced? Meanwhile, the fact that miRNAs and siRNAs could interact with each other adds another layer of complexity to this issue. Specifically, the altered expression levels of one might disrupt the existing miRNA-siRNA balance in cells and cause changes in the expression levels of the other by saturating the sRNA-induced silencing machinery since both miRNAs and siRNAs are utilizing an overlapped pathway in their biogenesis and metabolism processes [31–33].

To further explore the underlying mechanisms of viral resistance in plants, our study offers a new perspective as the first study that compares changes of siRNAs and miRNAs expression profiles between wild type and transgenic viral resistant plants. Illumina small RNA deep sequencing is applied onto both wild type and ihpRNA transgenic rice plants before and after RSV infection. Those siRNAs derived from ihpRNA insertion, novel miRNAs and known miRNAs are identified and compared. Our analyses suggest the accuracy of siRNA biogenesis is reduced in transgenic plants. We also find many known miRNAs involved in the antiviral process and their putative gene targets are predicted. Lastly, we discuss the potential mutual impact and interaction between siRNAs and miRNAs for future research.

## Materials and Methods

### Plant Material

Our RSV resistant transgenic rice plants (T4B1) were engineered with the ihpRNA strategy using *Oryza sativa* cv. Aichiasahi (AiA) as the background (Li et al., unpublished material). To trigger viral defensive processes in plants, each experimental seedling was inoculated with approximately five planthoppers. The viruliferous and virus-free planthoppers (mock group) were introduced respectively into the plant growth chambers at the three-leaf stage of rice for virus inoculation. After 48 h, the planthoppers were removed, and the inoculated seedlings were maintained under 14-h/10-h light/dark cycle at 25°C greenhouse condition until the total RNA was isolated. The leaf materials for the total RNA extraction were obtained from one young leaf per treatment. The young leaves were collected one week after the virus-induced syndromes appeared (i.e., 52 days after the inoculation) and the total RNAs were isolated subsequently.

### Small RNA sequencing and data pre-processing

Small RNA libraries were prepared with Illumina Small RNA library kit by following manufacturer’s protocol. Four libraries were obtained: AiA_VF and AiA_V represented wild type plants inoculated by virus-free insects and viruliferous insects respectively; similarly, T4B1_VF and T4B1_V represented the T_5_ of transgenic plants inoculated by virus-free and viruliferous insects respectively. Small RNA-Seq was conducted in Beijing Genomics Institute (BGI) by single-end sequencing. The four sRNA FASTQ files have been deposited in the NCBI Sequence Read Archive (study accession ID: SRP049382). The clean reads were obtained after trimming the sequencing adapters and filtering out the low quality reads. Clean reads ranging from 18–30 nucleotides in length (97% of total clean reads) were used for downstream analyses. Small RNA reads were then mapped to the rice genome to study their genomic origins and small RNA annotation by using SOAP [34] and custom scripts as previously described [35]. All clean sequence reads of siRNAs/miRNAs were summarized into unique sequence tags (denoted as siRNA/miRNA species) with expression information.

### siRNA identification and origin analysis

siRNA duplex sequences were identified by Tag2siRNA [35]. After normalization, siRNA species sequences supported by at least 3 reads were further selected. GSNAP [36] was used to map siRNA reads to RSV coat protein (RSVCP) reference sequence and rice genome (Osativa-193, downloaded from http://www.phytozome.net/) to determine the origin of siRNAs. Varied mismatch settings of mapping were used for biogenesis accruracy analysis.

### miRNA identification and miRNA expression profiling analysis

The novel miRNA and known miRNA were identified by Mireap (BGI: http://sourceforge.net/projects/mireap). To annotate known miRNA reads, all the clean reads are aligned to the rice miRNA precursor database (downloaded from www.mirbase.org). To study the known miRNA expression profiling, annotated miRNA species sequences with at least 3 supported reads were selected. The expression level changes for miRNAs were analyzed using DESeq [37].

### Target prediction

psRNATaget web server (http://plantgrn.noble.org/psRNATarget/) was used to predict the putative targets for specific siRNA and miRNA [38]. The major ihpRNA construct derived siRNAs and most dramatically changed miRNAs species were used as the query sequences. The MSU rice genome annotation version 7 was selected as a reference set. Maximum expectation was set to be 2 to obtain prediction results with a lower false positive rate, and other settings remained default.

## Results

### The small RNA profiles of the four datasets

**Figure 1:**
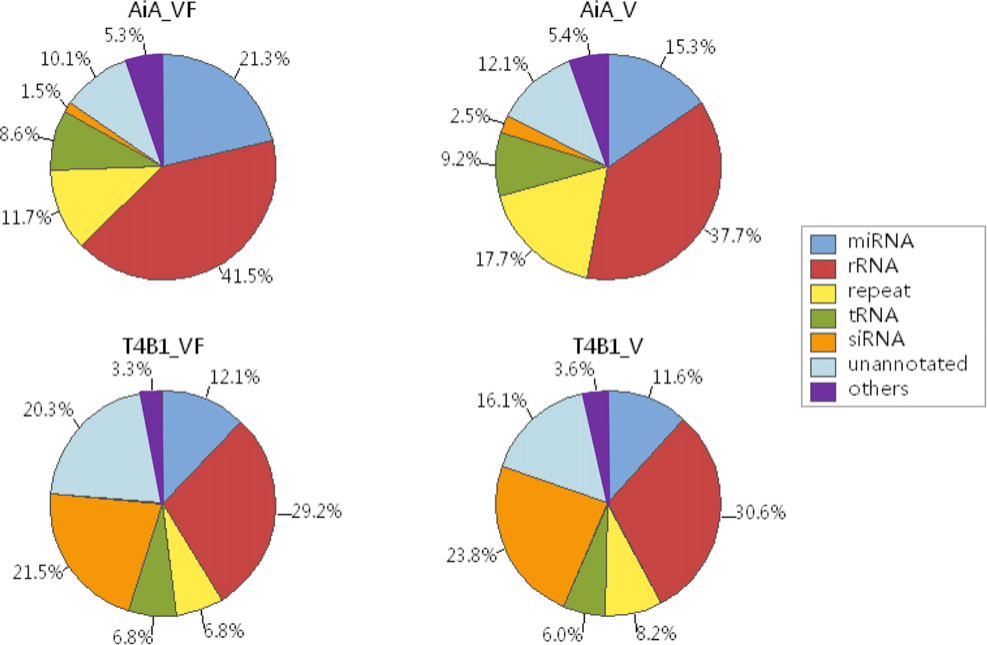
Pie charts of sRNA distribution for four datasets. All sRNAs are annotated to a specific category, including miRNA, siRNA, exon_sense, intron_sense, rRNA, repeat sequence, tRNA, unannotated sRNA and so on.

After filtering out the low quality reads and retaining reads of 18–30 nt in length, we obtained 10,393,417 clean reads for AiA_VF library, 12,934,262 for AiA_V, 10,422,352 for T4B1_VF and 14,094,201 for T4B1_V. The length distributions of small RNAs were shown in S1 Fig. It is clear that the majority of small RNA sequences for all 4 datasets were 20–25 nt in length. Those clean reads were then aligned to rice genome using SOAP. 91.4% (9,497,141 out of 10,393,417) of the total clean sRNA reads in AiA_VF library, 89.9% (11,629,382 out of 12,934,262) of the total clean reads in AiA_V, 59.4% (6,187,154 out of 10,422,352) of the total clean reads in T4B1_VF, and 61.4% (8,658,064 out of 14,094,201) of the total clean reads in T4B1_V were aligned to the rice genome successfully. All sRNAs reads that were mapped to the rice genome have been further annotated and categorized as or as a short fragment derived from miRNAs, siRNAs, rRNAs, tRNAs, and other un-annotated RNAs (see Fig. 1 for details).

### Analysis of the overall changes in siRNA expression levels

The T4B1 group generated more than 10 folds of the siRNA than AiA group (see Table 1). The emersion of specific siRNAs in transgenic plants demonstrated that the inserted ihpRNA construct could be recognized by siRNA machinery in host plants and was able to yield siRNAs. Those newly generated siRNAs repressed the expression of RSVCP gene hence impeded the viral replication process in rice. The increased expression of siRNAs in transgenic plants was demonstrated by northern blotting (Li et al. unpublished material). Moreover, varied expression levels of specific siRNAs were found among different segments of the RSVCP sequence, indicating a potential hotspot region for generating siRNAs for RNAi silencing in transgenic plants (Li et al., unpublished material).

**Table 1:** The total clean sRNA, siRNA and miRNA reads among 4 libraries.

	***Total clean sRNA Reads***	***Total siRNA reads (%)***	***Total miRNA reads (%)***
AiA_VF	10,393,417	153,884 (1.5%)	2,209,576 (21.3%)
AiA_V	12,934,262	323,476 (2.5%)	1,978,756 (15.3%)
T4B1_VF	10,422,352	2,242,150 (21.5%)	1,259,531 (12.1%)
T4B1_V	14,094,201	3,359,888 (23.8%)	1,636,496 (11.6%)

The percentage of miRNA/siRNA is calculated by dividing the total miRNA/siRNA reads with the total clean sRNA reads.

### The accuracy of siRNA biogenesis might be influenced in transgenic plants

**Figure 2:**
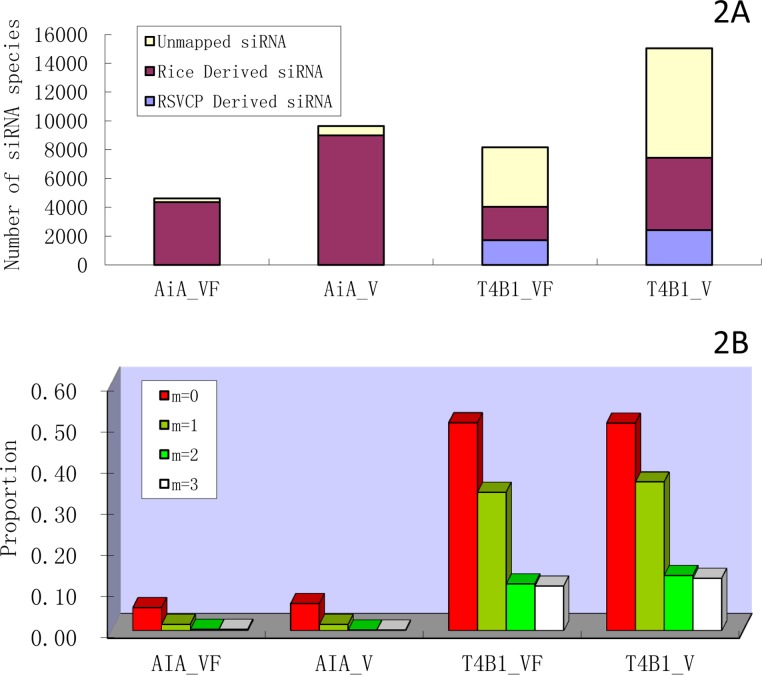
Identification of siRNA origin by mapping different miRNA species to rice genome and RSVCP sequence. A. Classification of siRNA among all four libraries when m = 0. Based on its origin, siRNA are classified into three groups, unmapped siRNA, rice derived siRNA and RSVCP derived siRNA. B. Un-mapped sequences analysis using GSNAP with different parameter settings among four datasets.

To further understand siRNA biogenesis mechanism, the origins of siRNAs in four datasets were analyzed. Firstly, GSNAP was used to align all different siRNA species to RSVCP sequence, allowing no mismatch (m = 0, see Fig. 2A). No siRNA species in AiA_VF and only one siRNA species in AiA_V was mapped perfectly to RSVCP gene within RSV reference sequence. For those siRNA species that cannot be mapped to RSVCP perfectly, they were further selected and aligned to rice genome using the same parameter setting (m = 0). It was found that around 95% of these RSV-unaligned siRNA species can be aligned to the rice reference genome. Those siRNAs that were mapped to rice genome, but not RSVCP, should be classified as endogenous siRNAs (or rice derived siRNAs) [39, 40]. As expected, in the two datasets of T4B1 (T4B1_VF and T4B1_V), a much greater proportion of siRNAs can be mapped with RSVCP gene sequence (21% for T4B1_VF and 16% for T4B1_V) and there is smaller proportion of endogenous siRNA derived from rice genome (28% for T4B1_VF and 33% for T4B1_V). Surprisingly, different proportions of unmapped siRNA species, which could neither map to rice genome nor RSVCP gene sequence, were found between the wild type and transgenic plants (5.5% in AiA group and 50% in T4B1 group, see Fig. 2A). This drastic difference led us to wonder where do those unmapped siRNAs come from. To study their origin, a similar mapping approach had been applied to map all four datasets to both rice genome and RSVCP sequence using gradually loosed parameter settings (i.e., 1, 2 or 3 mismatches). Consequently, the proportion of unmapped siRNAs of four datasets decreased accordingly. As shown in Fig. 2B, the T4B1 group contains much more un-mapped siRNA species in comparison with the AiA group. When the parameter setting (m = 3) is used, most siRNAs (>99%) in the AiA group found their origin either from rice genome or RSVCP sequence, and there was still 11% of siRNAs in the T4B1 group that could not find their origins (Fig. 2B). Therefore, it was speculated that the siRNA machinery is affected due to ihpRNA construct in transgenic plants (T4B1).

### Known miRNA profiling changes revealed potential function of several miRNA species

Although RSV-derived siRNAs might be the vital reason for this viral resistance in our case, the contribution of miRNA for RSV resistance cannot be ignored. In order to examine the expression profile changes of known miRNAs and to study the possible connection between miRNA profiling with the viral resistance, DESeq was used to compare miRNA expression levels among four libraries [28, 41]. Being mostly consistent with previous reports, the top ten highly expressed miRNAs species in our libraries were miR 156, 168, 167, 528, 166, 5794, 397, 820, 535 and 812, indicating their essential regulatory functions in plant viral defense for rice [26, 42]. Moreover, five out of six miRNAs (*i.e.*, miR 156, 159, 166, 167, 396) that are described to be highly expressed (>1000 reads supported) in Du’s paper [42] presented similar regulation patterns (either up-regulated or down-regulated) after RSV infection in our study.

The changes of known miRNA expression profiling were studied using DESeq from three aspects: the impact on expression profiling of known miRNA caused by the engineered ihpRNA construct was studied by comparing AiA_VF and T4B1_VF, the impact caused by viral infection in wild type plants was studied by comparing AiA_VF and AiA_V, and the impact caused by viral infection in transgenic plants was studied by comparing T4B1_VF and T4B1_V respectively. As described in Table 2, the expression levels of miR-528, 1318, 1432, 397, 1875, 408, 169 and 164 were dramatically changed (either up-regulated or down-regulated, P-value< 0.05) after transfecting ihpRNA construct, the expression levels of miR-399, 5156, 159, 5077, 1320, 444 and 396 were dramatically changed after RSV infection in wild type plants; and the expression levels of miR-5810, 159 and 164 were dramatically changed after RSV infection in T4B1 plants.

**Table 2:** The most dramatically differentially expressed miRNA species.

***miRNA name***	***FoldChange***	***Log2FoldChange***	***P-value***	***Padj***
WT_VF and WT_V				
MIR399	5.30863	2.40834	0.00521	0.54871
MIR5156	0.24364	-2.03720	0.00940	0.54871
MIR159	7.30883	2.86964	0.01220	0.54871
MIR5077	12.95807	3.69578	0.01379	0.54871
MIR1320	0.21677	-2.20574	0.03953	0.63538
MIR444	4.05349	2.01916	0.04204	0.63538
MIR396	9.39446	3.23181	0.04568	0.63538
T4B1_VF and T4B1_V				
MIR5801	0.06446	-3.95539	0.00084	0.16771
MIR159	6.02189	2.59022	0.01379	0.83509
MIR164	5.82169	2.54144	0.03000	0.83509
WT_VF and T4B1_VF				
MIR528	7.79491	2.96253	0.00002	0.00385
MIR1318	3.19742	1.67691	0.01325	0.73245
MIR1432	3.19742	1.67691	0.01325	0.73245
MIR397	3.05039	1.60899	0.01619	0.73245
MIR1875	0.28156	-1.82849	0.01951	0.73245
MIR408	2.99918	1.58457	0.02576	0.73245
MIR169	0.29728	-1.75011	0.03504	0.79880
MIR164	3.31047	1.72704	0.03613	0.79880

Foldchange presents fold change from the first to the latter condition. Log2FoldChange presents the logarithm (to basis 2) of the fold change. P-value and Padj is provided by DESeq.

### RSV infection related novel miRNAs were not found

**Figure 3:**
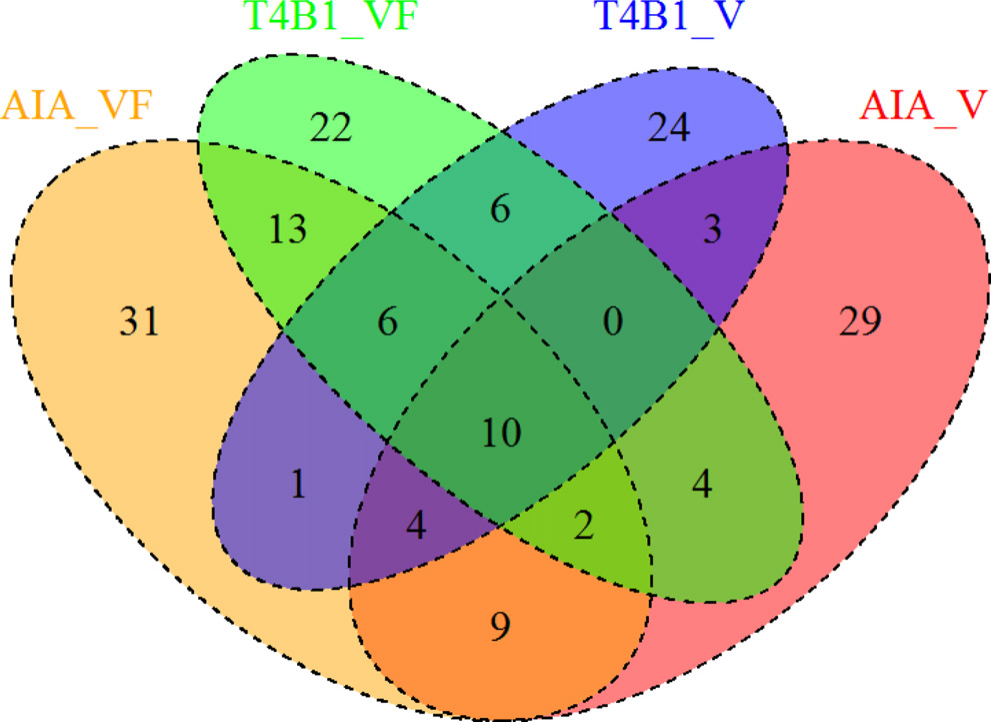
The venn diagram for the shared novel miRNAs among four libraries.

To study the effect of novel miRNAs in transgenic rice plants, putative novel miRNAs (see S1 Table) were predicted. Among the four libraries, the numbers of putative novel miRNA species were 76, 61, 63 and 54 respectively. All of these novel miRNAs were supported by 3 ~ 205 sequence reads. To test whether or not the novel miRNAs emersion was correlated with viral infection as previously described [26], all novel miRNA species were analyzed and compared to identify the common and unique ones among different datasets (Fig. 3). It turned out that there was no specific novel miRNA species had been induced either by transfecting ihpRNA construct or RSV infection in both wild-type or transgenic plants.

### Target prediction

To study the impact of RSV-derived siRNAs in transgenic rice plants besides their original function of silencing RSV-CP genes, the top 50 highly expressed RSV-derived siRNAs from T4B1_VF were selected as input query sequences for psRNAtarget web server [38] to predict putative targets. Those RSV-derived siRNAs were fairly representative (34.1% of the total siRNA reads in T4B1_VF). Predicted target genes were found to be mainly involved in regulating disease resistance proteins and zinc finger protein (S2 Table). The most differently expressed miRNAs between AiA_VF with T4B1_VF (miRNA 528, 1318, 1432, 397, 1875, 408, 169 and 164, see Table 2) were also selected to study their biological importance in transgenic plants. Target predictions of these miRNAs showed that they affected a number of genes involving in protein metabolism and DNA replication processes (S3 Table).

## Discussion

As shown in Table 1, total reads number of siRNA in the transgenic plants (T4B1_VF and T4B1_V) is approximately 12 times that of the wild type plants (AiA_VF and AiA_V). The drastic emersion of specific siRNAs reads in transgenic plants supported that the inserted ihpRNA construct could be recognized by host plant siRNA machinery that yields siRNAs to complementarily pair with RSVCP gene transcripts. As a consequence, viral replication is suppressed in transgenic rice. By aligning the siRNA species to RSVCP sequence with perfect alignment, the results show that T4B1 group has an average of 18.5% siRNA sequence species are derived from that RSVCP segment (see Fig. 2A). To note, there is also a big proportion of endogenous siRNAs, derived from rice genome, in both AiA and T4B1 groups (see Fig. 2A). The rest of siRNA sequences which can neither map with the rice genome nor the RSVCP have an unclear original source but the proportional difference of them between AiA group and T4B1 group is huge (5.5% in AiA group and 50% in T4B1 group). The unmapped sequences are further analyzed by using gradually loosed parameters with more mismatches. When allowing maximum 3 mismatches for alignment, more than 99% of siRNAs in AiA group found their origin either from rice genome or RSVCP, and the proportion of unmapped siRNA in T4B1 group is dramatically decreased to 11%. Alignment parameter (m = 3) is not further loosed because little decrease of proportion of unmapped siRNA species is found at m = 4. Actually, the result, with alignment parameter setting at m = 2, is very close to what is at m = 3. On the other hand, allowing 4 mismatches for short siRNA reads will result in false positive alignments. The result of progressively relaxed alignment (Fig. 2B) leads to the assumption that the artificial ihpRNA construct has influenced the homeostatic environment for siRNA biogenesis in host cells. Thus, the accuracy of siRNA production is likely to be harmed when the siRNA machinery is excessively used.

Previous research claims the possible role miRNA plays in the process of self-defensive response to virus [15]. Regarding to the known miRNA expression profiling change, as shown in Table 2, eight miRNA species are dramatically varied after transfecting ihpRNA construct; seven miRNA species are dramatically changed after RSV infection in wild type plants and three miRNA species are dramatically changed in mutant plants. Unfortunately, the difference of most dramatically changed miRNAs did not reach the statistical significance when using the Padj-value, except miRNA 528 (Padj-value = 0.00385) when comparing AiA_VF and T4B1_VF. Since only one replicate per treatment/library has been utilized in our study, the power of statistics in our study that evaluates the true role that miRNAs play in viral resistance process is reduced. To explain the dramatically altered expression level of eight miRNA species between AiA_VF and T4B1_VF, an alignment test is carried out to see any possible direct interaction between the newly produced, RSV-derived siRNAs with known miRNA precursor sequences using BLAST [43]. According to our analysis (result is not shown), those miRNAs (miRNA precursors) are not affected via direct pairing-wise manner by newly produced RSV-derived siRNAs. Hence the inserted ihpRNA construct influence the expression of those miRNA species with an unclear reason. In addition, a recent research suggested miRNA*s sequence also played a role in viral defensive process [42] makes the virus induced regulation of miRNA more mystery. In terms of novel miRNAs, our result is contrary to previous report, in which Guo et al have reported that seven novel rice miRNAs were produced related to RSV infection [26]. However in our research, there is no apparent evidence of supporting any novel miRNAs related with RSV infection, as shown in Fig. 3. Thus, those novel miRNAs are more likely due to the sequencing technique and methodology improvement over the time, without significant differences between any two treatments/libraries.

The crosstalk of siRNA and miRNA is also briefly examined in our study. Liang et al have reported that manually induced siRNAs can alter the expression level of many miRNA through competing Ago2 loading site [44]. Limited abundance of the posttranscriptional machineries in host cell causes the saturation effect, when exogenous sRNAs are generated. This saturation effect results in the fact that mRNA targets of endogenous miRNAs are normally up-regulated due to repression of miRNAs [40], although some evidence are implying two distinct pools of AGO1 protein are specifically utilized by miRNA and siRNA separately in *Arabidopsis* [31]. On the other hand, the posttranscriptional regulation effect of miRNAs and siRNAs on individual transcript target is alleviated when there is an overwhelming population of predicted transcript targets [33]. In our case, as the proportion of siRNAs increased from AiA to T4B1 plants (1.5% to 21.5%), the proportion of miRNA dramatically decreased correspondingly (21.3% to 12.1%) by comparing AiA_VF with T4B1_VF (Table 1). The result suggested that siRNAs became the primary product of RISC system after ihpRNA was transgenetically inserted, and the miRNA/siRNA machinery might be overused and thus repress the yielding of miRNAs in rice. The accuracy of miRNA production is also compared among four datasets (see S4 Table, the known miRNAs SNPs for four datasets were provided by Mireap). Unlike the siRNA, the SNP percentages of miRNAs maintain at a similar level among four libraries. Our result provides evidence stating that the accuracy of siRNA biogenesis is affected by ihpRNA construct while the accuracy of miRNA machinery remains unaffected.

## Conclusions

Collectively, our results provide the first research of the siRNA and known miRNA profiling changes in an ihpRNA engineered viral-resistant plant using high-throughput sequencing. The siRNA derived from ihpRNA construct is determined as an important contributor to the antivirual resistance in plants. The accuracy of siRNA biogenesis machinery might be influenced if excessively used, but the accuracy of miRNA biogenesis machinery seems not to be affected. In addition, our data analysis does not suggest any novel miRNAs involved in the process of RSV infection. We have also assessed the potential impact of differentially expressed miRNAs due to the insertion of ihpRNA construct or the RSV infection by predicting their targeting genes. In sum, this research will facilitate our understanding of the regulation and function of miRNAs and siRNAs in transgenic plants.

## Supporting Information

S1 Fig The length distribution of total sRNA reads among four datasets. (DOCX)

S1 Table The sequences of the predicted novel miRNAs among four datasets. (DOCX)

S2 Table The predicted targets of the top 50 most expressed RSV-derived siRNAs in T4B1 datasets by psRNAtarget. (DOCX)

S3 Table The predicted targets of the top known miRNAs with the greatest changes between AiA_VF and T4B1_VF datasets. (DOCX)

S4 Table The SNP ratio of miRNAs among four datasets. Normal count represents the population of miRNA reads without SNP. SNP count presents the population of miRNA reads with SNP (mismatches) from their miRNA precursors. The ratio is calculated by dividing SNP count with normal count.
(DOCX)
