# Supplementary figures and images for: Alterations in SiRNA and MiRNA Expression Profiles Detected by Deep Sequencing of Transgenic Rice with SiRNA-Mediated Viral Resistance
Source: PLoS One. 2015 Jan 5;10(1):e0116175. doi: 10.1371/journal.pone.0116175 (PMC4283965; doi:10.1371/journal.pone.0116175)

**
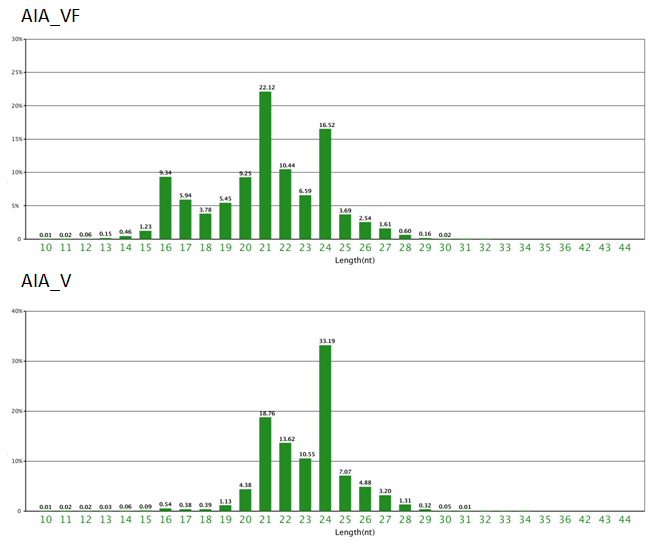
**

**
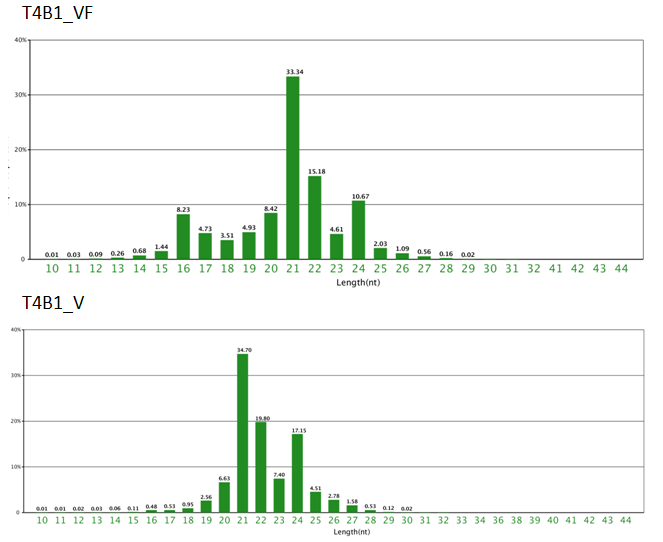
**

**Figure S1. The length distribution of total sRNA reads among four datasets.**

Supplement: S1 Fig — (DOCX) [file pone.0116175.s001.docx]
