# Supplementary material for: Alterations in SiRNA and MiRNA Expression Profiles Detected by Deep Sequencing of Transgenic Rice with SiRNA-Mediated Viral Resistance
Source: PLoS One. 2015 Jan 5;10(1):e0116175. doi: 10.1371/journal.pone.0116175 (PMC4283965; doi:10.1371/journal.pone.0116175)
Supplement: S1 Table — (DOCX) [file pone.0116175.s002.docx]

**Table S1. The sequences of the predicted novel miRNAs among four datasets.**

| **Novel miRNA Sequence** | | | |
| --- | --- | --- | --- |
| **AIA_VF** | **AIA_V** | **T4B1_VF** | **T4B1_V** |
| AGGGGATTTGCAATTATAACACT | TCGGGGAAAGGGGAAGGGGAA | TTTGCAATTTGGGCTAGTGGC | GGGAGTATTATGAAAGGAGGTTA |
| TCGGACTCTCGGCGGCGCTCG | TTTGCAATTTGGGCTAGTGGC | CTTTGAGTAGGGTCTAAACAGAG | TTTGCAATTTGGGCTAGTGGC |
| TAACGAGCAATGATTCAGGCAG | TTCATGCGCACATTATTAACTG | TGGACAATGGTTACGAGTAAT | CTTTGAGTAGGGTCTAAACAGAG |
| TTTGCAATTTGGGCTAGTGGC | CTTTGAGTAGGGTCTAAACAGAG | TCTGAAAACTAAAGAAGGGCA | CGGTGGCAGCATATTTGCATT |
| CTTTGAGTAGGGTCTAAACAGAG | GCCGACCTAGCTCAGTGGTAG | TGGACAATGGTTACGAGTAAT | AAGGAAATTGAGACGCTGGCATA |
| GTGTTCGGAAGAGCTAGCAGTGG | TGGCGAGTAGGATCCACGTGGAC | TCTGAAAACTAAAGAAGGGCA | TTAATCGTCGATCAGGCACAA |
| CGGTGGCGGACTGACGACGGC | TCGAAGATAGAAGAGCCCAGA | TCGACCACTACGAGCACGCCT | TCGATGCAGTCCTCGATGTCG |
| TTAATCGTCGATCAGGCACAA | ATAGCTTGCACTGTGAGAGGCCT | TGCTCCGGATATTATGGCATG | TGCTCCGGATATTATGGCATG |
| TCGATGCAGTCCTCGATGTCG | TGGACAATGGTTACGAGTAAT | ATGCAGTCCTCGATGTCGTAG | TTACGAACAGAAGCTCTTATT |
| TGCTCCGGATATTATGGCATG | GTAGGGGATGTAGCTCAGATGGT | GCGACTGGGACGGGGACGGC | CGGCGTCGTCTAGGCCGAGCGGC |
| ATAAGTTTTGGAAGGGTTTAT | TTAATCGTCGATCAGGCACAA | TCAGAATTTGGGAAGCGGTAC | TTACGAACAGAAGCTCTTATT |
| CAAGGGATAGATCGATTGCAATA | ATCGACCACTACGAGCACGCC | TTATAATGTAGAACAGAGGGA | TAATTGCTCTATTAGGATATC |
| AAGGAGAGGGGGAAGAGAAAG | TCGATGCAGTCCTCGATGTCG | GTTTTCGGTGGTATGTTGGCAAT | TGAATGGGATTCGTTTGGGCA |
| GCGACTGGGACGGGGACGGC | TGCTCCGGATATTATGGCATG | TAATTGCTCTATTAGGATATC | ACTGATTTGGTGGTTGACATG |
| CGGCGTCGTCTAGGCCGAGCGG | GGGGCCCCTCGCCGGCGCGCGTG | ATTTGATTTGTCGCGAGCAAT | GGGAGTATTATGAAAGGAGGTTA |
| TTATAATGTAGAACAGAGGGA | CGGCGTCGTCTAGGCCGAGCGG | AAGCTTATTGACGGTCCAGTGC | AAGCTTATTGACGGTCCAGTGCT |
| AACAAGTCTGGGTGGTGTAGTTG | TTATAATGTAGAACAGAGGGA | GATTCAGATTGGGTGACATAG | GATTCAGATTGGGTGACATAG |
| CGGCTGGCGACGGGGACGGCG | AAACGATGAGACGGAAAAGTA | CACGGAGAGGGAAAAAGGCGC | TCTGACGTAGAACTAGAGGGAC |
| TAATTGCTCTATTAGGATATC | GTTTTCGGTGGTATGTTGGCAAT | GACGGCTCTCAGACGAACGGGTG | TGATGACGTGTTAGAAATGTA |
| AAGCTTATTGACGGTCCAGTG | TAATTGCTCTATTAGGATATC | AAGGAAGGAGGATTTTCAATGCG | TGGATGCCTGACCAAAGCGAC |
| ATTTGAATAGGAAAATCTGCATA | AAGCTTATTGACGGTCCAGTGCT | AGGCTGTGGAGAGATGGCTGA | GCTGGAGTAGCTCAGATGGT |
| TGAACAAGTAAAACGTGAGCT | CGGCTGGCGACGGGGACGGCG | TGGATGCCTGACCAAAGCGAC | TTTGCAATTTGGGCTAGTGGC |
| CGGCTGGCGACGGGGACGGCG | AAGGGGCGCTTACTGAGAGTT | GCGACTGGGACGGGGACGGC | TTTCTTGACTCGGGATGACTAG |
| TCCAATGCAGATACTGAGACC | TGGTGTGAGAGAATGAAGCTG | GAGGGACTAGGGGAGGGAGGAG | TTTGAGGATGGAGGTAGTATC |
| GTGTTCGGAAGAGCTAGCAGTGG | TCTCTTCGATCGATCTAGGGTT | GGGTACTGACAGTTGTGGCTTG | GGGAGTATTATGAAAGGAGGTTA |
| TCGGAACAGAGGGAATAGGAG | AATGGTTGTATTGGTCGTACGGA | GCTGGAGTAGCTCAGATGGT | TAGAGCTTCGTGCTGATAAC |
| GCGACTGGGACGGGGACGGC | TACGAGAGATGGGAAAAGACAAC | AGTAAATTTGGTGACGTGGAAGA | GTGGATTTGGGGGGTGGGGGTGT |
| AGAATCGGCGAGTATACTTTA | ATAGCTTGCACTGTGAGAGGCCT | TCTGAAAACTAAAGAAGGGCA | TAAGGACAATAGGAATGGGAGGC |
| GCTGGAGTAGCTCAGATGGT | TTTGCAATTTGGGCTAGTGGC | TCTGAAAACTAAAGAAGGGCA | TCACGGGATAGAGATATGGTA |
| TACGAGAGATGGGAAAAGACAAC | TTTCTTGACTCGGGATGACTAG | TCAGAATTTGGGAAGCGGTAC | CGGCTGGCGACGGGGACGGCG |
| CAAGGGATAGATCGATTGCAATA | TATTTGAGGATGGAGGTAGTA | TTTGCAATTTGGGCTAGTGGC | AGGATTTGTTGAACTATAGGGTA |
| TTTGCAATTTGGGCTAGTGGC | TTAGGGAAGATTATTTAGGACA | TTTCTTGACTCGGGATGACTAG | TTACGAACAGAAGCTCTTATT |
| TTTCTTGACTCGGGATGACTAG | CGGCTGGCGACGGGGACGGCG | TTTGAGGATGGAGGTAGTATC | GATTCAGATTGGGTGACATAG |
| TTTGAGGATGGAGGTAGTATC | AGGATTTGTTGAACTATAGGGTA | TAAGGACAATAGGAATGGGAGGC | TGCGAAGTAGAGATGCCGACT |
| ATATCTTATAAGGTCGCGTGT | AGCGGGAGACACGGAGGAGCG | CACGGAATGGAAGAGCGAGAC | TTTGTTGCAATTTGGACTACC |
| AGAGCTTCGTGCTGATAACG | TTTGTTGCAATTTGGACTACC | TGCGAAGTAGAGATGCCGACT | TTACGAACAGAAGCTCTTATT |
| ATAAGTGATTTGGTTGAATTGGA | GGTTTTTATTATGATCCGGAACA | TTTGTTGCAATTTGGACTACC | TAGTTTCTGCCACAAGATCGTG |
| TAAGGACAATAGGAATGGGAGGC | AATCTCAGTGGTCGTGCCGGAGT | ACCCCCCTTTGCCGGCGCGCGC | TCTGGAGAAGACGATTGAGCTA |
| TTAGGGAAGATTATTTAGGACA | ATAAAATCTATGGTCTCTGTTTC | TAAAATGGCATCCTCTCTTTC | ATTTAGCATTAGGATCAGACGT |
| CGGCTGGCGACGGGGACGGCG | TGTGCTGCTGCTGCGAGACGTGT | TTTGGTGCTGAACAGAAGGGTC | GATTCAGATTGGGTGACATAG |
| AGGATTTGTTGAACTATAGGGTA | AAATATATTTGATGTTGGGAC | TAGTTTCTGCCACAAGATCGTG | TACGGCCAACTGATTCATGCAT |
| AATGGATTTTACATGGATGG | TGGACGGACACTAAGATGGAAT | CGGCCATGGACGACGACGACGG | TCAGCACGGATACATTATTTT |
| CACGGAATGGAAGAGCGAGAC | TCTGGAGAAGACGATTGAGCTA | TCTGAAAACTAAAGAAGGGCA | TTACGAACAGAAGCTCTTATT |
| TGCGAAGTAGAGATGCCGACT | TCATGTATTAATGAACGGAGG | TCTGGAGAAGACGATTGAGCTA | TTGGATTGGCTGGTTAGATTAT |
| GCTTATTGTCTCGTTTTTGTTCT | ACTCGGGATGTAATGTGCATGG | GATTCAGATTGGGTGACATAG | AGCTAAGAGCTGAAACTCGTGAA |
| TTTGTTGCAATTTGGACTACC | TAGGAAATCAATTTTAGGCTA | TTGGATTTGGATAGAAGGGTA | GAGTAGATTTTAGGAAGGCAG |
| ACCCCCCTTTGCCGGCGCGCGC | TTGGATTTGGATAGAAGGGTA | TAAAATGGCATCCTCTCTTTC | TGGACAAAAGTGAAGAAATGCC |
| ATAAGTTTTGGAAGGGTTTAT | TTGGATTGGCTGGTTAGATTAT | TCTGAAAACTAAAGAAGGGCA | GATTCAGATTGGGTGACATAG |
| CGGCGATGTGGGGGAGGCGCG | TAAAAGAGAACTGAGGGAGTAA | AAGAGTTGCTTCTGTCGTCTGTC | TTACGAACAGAAGCTCTTATT |
| TAGTTTCTGCCACAAGATCGTG | AGAGTAGATTTTAGGAAGGCA | AGTAAATTTGGTGACGTGGAAGA | TTCTCACTTTGGACTAGGTAT |
| CGGCGATGTGGGGGAGGCGCG | AAAATTGGGGATGTCTGTCAGA | TCTGAAAACTAAAGAAGGGCA | TGTGTAGCCACATTGTAAGGG |
| ATAAGTTTTGGAAGGGTTTAT | AAATTCTTGATGTTTTGGGTG | GTGAGCGAGACCGAATGGCT | ATAATTTGGTAGAGGACAATTC |
| TCTGAAAACTAAAGAAGGGCA | AAACTAATCTAGGATTCGGTG | TGGACAAAAGTGAAGAAATGCC | TTGGTAAGGCGAAAATTGGCAT |
| ATGTTCACTTCTACTCGTCATC | ACTCGCTGGATTGATCCCAAA | GATTCAGATTGGGTGACATAG | TCATGGCGCTCACGGTTAGATC |
| TCTGGAGAAGACGATTGAGCTA | TTAGGTGGTTCTCGGAAGAAG | TCTGAAAACTAAAGAAGGGCA |  |
| TCATGTATTAATGAACGGAGG | TTTAAATTGCAGATTTAGGTT | TTAGGTGGTTCTCGGAAGAAG |  |
| ACTCGGGATGTAATGTGCATGG | AGATCGGAGCGCGGCCTCGCCA | TGGACAAAAGTGAAGAAATGCC |  |
| TAGGAAATCAATTTTAGGCTA | TTCTCACTTTGGACTAGGTAT | AGGGGTTGTTTGTGATAGGGGGA |  |
| TGCCAGAGGAGAACTGCACTG | TAATTGTACGTGGAAGTTGGA | GGGTACTGACAGTTGTGGCTTG |  |
| TCAGCACGGATACATTATTTT | TTGGTAAGGCGAAAATTGGCAT | TGTGTAGCCACATTGTAAGGG |  |
| AGGACGGGTACGACTACGGCA | TCATGGCGCTCACGGTTAGATC | AGGTGGACCATATTTTTAGCTGT |  |
| TCCTTTAACCTTGTAAACTGCAG |  | TTGGTAAGGCGAAAATTGGCAT |  |
| ATAAGTTTTGGAAGGGTTTAT |  | TCATGGCGCTCACGGTTAGATC |  |
| GTGAGCGAGACCGAATGGCT |  |  |  |
| ACGAGGTTGGTTTATTTTGGA |  |  |  |
| TTGTTTATAGACTGGACAGGCG |  |  |  |
| AAATTCTTGATGTTTTGGGTG |  |  |  |
| TAAGGATACCAAGCATATGATG |  |  |  |
| TTAGGTGGTTCTCGGAAGAAG |  |  |  |
| CAAGGGATAGATCGATTGCAATA |  |  |  |
| TGTGTAGCCACATTGTAAGGG |  |  |  |
| AAGGTAGCTATATTTCGGGAC |  |  |  |
| AACGAAAGAGAGATAGGGTCCT |  |  |  |
| TAATTGTACGTGGAAGTTGGA |  |  |  |
| TTGGTAAGGCGAAAATTGGCAT |  |  |  |
| TCATGGCGCTCACGGTTAGATC |  |  |  |
