# Supplementary material for: Alterations in SiRNA and MiRNA Expression Profiles Detected by Deep Sequencing of Transgenic Rice with SiRNA-Mediated Viral Resistance
Source: PLoS One. 2015 Jan 5;10(1):e0116175. doi: 10.1371/journal.pone.0116175 (PMC4283965; doi:10.1371/journal.pone.0116175)
Supplement: S2 Table — (DOCX) [file pone.0116175.s003.docx]

**Table S2. The predicted targets of the top 50 most expressed RSV-derived siRNAs in T4B1 datasets by psRNAtarget.**

| miRNA_Acc. | Target_Acc. | E | UPE | Inhibition | Target_Desc. |
| --- | --- | --- | --- | --- | --- |
| t0000020 | LOC_Os07g25240.1 | 2 | 18.728 | Cleavage | cDNA\|transposon protein, putative, CACTA, En/Spm sub-class |
| t0000020 | LOC_Os02g25380.1 | 2 | 12.049 | Cleavage | cDNA\|transposon protein, putative, CACTA, En/Spm sub-class, expressed |
| t0000020 | LOC_Os05g27850.1 | 2 | 12.641 | Cleavage | cDNA\|transposon protein, putative, CACTA, En/Spm sub-class, expressed |
| t0000020 | LOC_Os03g54030.1 | 2 | 12.406 | Cleavage | cDNA\|transposon protein, putative, CACTA, En/Spm sub-class, expressed |
| t0000037 | LOC_Os11g13940.1 | 2 | 17.498 | Cleavage | cDNA\|NBS-LRR disease resistance protein, putative, expressed |
| t0000037 | LOC_Os10g02980.1 | 2 | 20.196 | Translation | cDNA\|formin, putative, expressed |
| t0000038 | LOC_Os03g61700.1 | 2 | 18.392 | Cleavage | cDNA\|expressed protein |
| t0000082 | LOC_Os06g49380.5 | 2 | 16.26 | Translation | cDNA\|NBS-LRR disease resistance protein, putative, expressed |
| t0000082 | LOC_Os06g49380.6 | 2 | 16.26 | Translation | cDNA\|NBS-LRR disease resistance protein, putative, expressed |
| t0000082 | LOC_Os06g49380.3 | 2 | 16.26 | Translation | cDNA\|NBS-LRR disease resistance protein, putative, expressed |
| t0000082 | LOC_Os06g49380.4 | 2 | 16.26 | Translation | cDNA\|NBS-LRR disease resistance protein, putative, expressed |
| t0000082 | LOC_Os06g49380.1 | 2 | 16.26 | Translation | cDNA\|NBS-LRR disease resistance protein, putative, expressed |
| t0000082 | LOC_Os06g49380.2 | 2 | 16.26 | Translation | cDNA\|NBS-LRR disease resistance protein, putative, expressed |
| t0000090 | LOC_Os10g01920.1 | 2 | 9.099 | Cleavage | cDNA\|transferase family protein, putative, expressed |
| t0000121 | LOC_Os11g13940.1 | 2 | 17.498 | Cleavage | cDNA\|NBS-LRR disease resistance protein, putative, expressed |
| t0000121 | LOC_Os10g02980.1 | 2 | 20.196 | Translation | cDNA\|formin, putative, expressed |
| t0000135 | LOC_Os03g41110.1 | 2 | 14.063 | Cleavage | cDNA\|ZOS3-14 - C2H2 zinc finger protein, expressed |
| t0000157 | LOC_Os11g13940.1 | 2 | 18.031 | Cleavage | cDNA\|NBS-LRR disease resistance protein, putative, expressed |
| t0000157 | LOC_Os10g02980.1 | 2 | 19.655 | Cleavage | cDNA\|formin, putative, expressed |
| t0000170 | LOC_Os10g20750.1 | 2 | 14.75 | Cleavage | cDNA\|retrotransposon protein, putative, unclassified, expressed |
| t0000170 | LOC_Os10g02040.1 | 2 | 17.535 | Cleavage | cDNA\|peroxidase precursor, putative, expressed |
| t0000170 | LOC_Os10g02040.2 | 2 | 17.535 | Cleavage | cDNA\|peroxidase precursor, putative, expressed |
| t0000170 | LOC_Os10g42410.1 | 2 | 5.765 | Translation | cDNA\|zinc-binding protein, putative, expressed |
| t0000170 | LOC_Os10g42410.3 | 2 | 5.765 | Translation | cDNA\|zinc-binding protein, putative, expressed |
| t0000170 | LOC_Os10g42410.2 | 2 | 5.765 | Translation | cDNA\|zinc-binding protein, putative, expressed |
| t0000211 | LOC_Os06g44620.1 | 2 | 15.612 | Translation | cDNA\|AMP-binding domain containing protein, expressed |
| t0000211 | LOC_Os12g14740.1 | 2 | 24.107 | Translation | cDNA\|retrotransposon protein, putative, Ty1-copia subclass, expressed |
| t0000269 | LOC_Os10g01920.1 | 2 | 8.411 | Cleavage | cDNA\|transferase family protein, putative, expressed |
| t0000283 | LOC_Os10g01920.1 | 2 | 9.099 | Cleavage | cDNA\|transferase family protein, putative, expressed |

**E:** the false positive prediction rate.

**UPE:** the allowed maximum energy to unpair the target site.
