# Supplementary material for: Alterations in SiRNA and MiRNA Expression Profiles Detected by Deep Sequencing of Transgenic Rice with SiRNA-Mediated Viral Resistance
Source: PLoS One. 2015 Jan 5;10(1):e0116175. doi: 10.1371/journal.pone.0116175 (PMC4283965; doi:10.1371/journal.pone.0116175)
Supplement: S3 Table — (DOCX) [file pone.0116175.s004.docx]

**Table S3. The predicted targets of the top known miRNAs with the greatest changes between AiA_VF and T4B1_VF datasets.**

| miRNA_Acc. | Target_Acc. | E | UPE | Inhibition | Target_Desc. |
| --- | --- | --- | --- | --- | --- |
| osa-miR399d | LOC_Os05g48390.1 | 1 | 17.119 | Cleavage | cDNA\|ubiquitin conjugating enzyme protein, putative, expressed |
| osa-miR399d | LOC_Os05g45350.2 | 2 | 24.843 | Cleavage | cDNA\|dnaJ domain containing protein, expressed |
| osa-miR5156 | LOC_Os02g06215.1 | 2 | 23.596 | Cleavage | cDNA\|RNA-directed DNA polymerase, putative, expressed |
| osa-miR5156 | LOC_Os02g06210.1 | 2 | 23.596 | Cleavage | cDNA\|phytosulfokine receptor precursor, putative, expressed |
| osa-miR159a.1 | LOC_Os01g12700.1 | 2 | 14.208 | Cleavage | cDNA\|MYB family transcription factor, putative |
| osa-miR159a.1 | LOC_Os05g41166.1 | 2 | 13.577 | Cleavage | cDNA\|MYB family transcription factor, putative, expressed |
| osa-miR1320-5p | LOC_Os12g42220.1 | 2 | 13.651 | Cleavage | cDNA\|expressed protein |
| osa-miR444e | LOC_Os02g49840.1 | 0 | 18.98 | Cleavage | cDNA\|OsMADS57 - MADS-box family gene with MIKCc type-box, expressed |
| osa-miR444e | LOC_Os02g49840.4 | 0 | 18.98 | Cleavage | cDNA\|OsMADS57 - MADS-box family gene with MIKCc type-box, expressed |
| osa-miR444e | LOC_Os02g49840.2 | 0 | 18.98 | Cleavage | cDNA\|OsMADS57 - MADS-box family gene with MIKCc type-box, expressed |
| osa-miR444e | LOC_Os02g49840.3 | 0 | 19.734 | Cleavage | cDNA\|OsMADS57 - MADS-box family gene with MIKCc type-box, expressed |
| osa-miR444e | LOC_Os08g33488.1 | 0 | 18.822 | Cleavage | cDNA\|OsMADS23 - MADS-box family gene with MIKCc type-box, expressed |
| osa-miR444e | LOC_Os08g06510.1 | 0.5 | 18.819 | Cleavage | cDNA\|zinc finger, C3HC4 type domain containing protein, expressed |
| osa-miR444e | LOC_Os08g33479.1 | 1 | 18.435 | Cleavage | cDNA\|expressed protein |
| osa-miR444e | LOC_Os02g36924.1 | 2 | 17.063 | Translation | cDNA\|OsMADS27 - MADS-box family gene with MIKCc type-box, expressed |
| osa-miR444e | LOC_Os04g38780.1 | 2 | 19.345 | Translation | cDNA\|transcription factor, putative, expressed |
| osa-miR444e | LOC_Os04g51350.1 | 2 | 17.504 | Cleavage | cDNA\|pentatricopeptide, putative, expressed |
| osa-miR396i | LOC_Os06g10310.1 | 0 | 17.97 | Cleavage | cDNA\|growth regulating factor protein, putative, expressed |
| osa-miR396i | LOC_Os06g02560.2 | 0 | 17.86 | Cleavage | cDNA\|growth-regulating factor, putative, expressed |
| osa-miR396i | LOC_Os04g51190.3 | 0 | 20.242 | Cleavage | cDNA\|growth-regulating factor, putative, expressed |
| osa-miR396i | LOC_Os04g51190.1 | 0 | 20.242 | Cleavage | cDNA\|growth-regulating factor, putative, expressed |
| osa-miR396i | LOC_Os04g51190.2 | 0 | 20.242 | Cleavage | cDNA\|growth-regulating factor, putative, expressed |
| osa-miR396i | LOC_Os02g47280.2 | 0 | 24.559 | Cleavage | cDNA\|growth-regulating factor, putative, expressed |
| osa-miR396i | LOC_Os06g02560.1 | 0 | 17.86 | Cleavage | cDNA\|growth-regulating factor, putative, expressed |
| osa-miR396i | LOC_Os06g02560.3 | 0 | 17.86 | Cleavage | cDNA\|growth-regulating factor, putative, expressed |
| osa-miR396i | LOC_Os02g47280.1 | 0 | 24.559 | Cleavage | cDNA\|growth-regulating factor, putative, expressed |
| osa-miR396i | LOC_Os02g53690.1 | 0 | 17.56 | Cleavage | cDNA\|growth regulating factor protein, putative, expressed |
| osa-miR396i | LOC_Os03g47140.1 | 0 | 14.61 | Cleavage | cDNA\|growth regulating factor protein, putative, expressed |
| osa-miR396i | LOC_Os04g24190.1 | 1 | 24.31 | Cleavage | cDNA\|growth-regulating factor 11, putative, expressed |
| osa-miR396i | LOC_Os04g48510.1 | 1.5 | 20.501 | Cleavage | cDNA\|growth regulating factor protein, putative, expressed |
| osa-miR396i | LOC_Os03g51970.1 | 1.5 | 24.155 | Cleavage | cDNA\|growth-regulating factor, putative, expressed |
| osa-miR396i | LOC_Os12g29980.2 | 1.5 | 15.862 | Cleavage | cDNA\|growth regulating factor protein, putative, expressed |
| osa-miR396i | LOC_Os12g29980.1 | 1.5 | 15.862 | Cleavage | cDNA\|growth regulating factor protein, putative, expressed |
| osa-miR396i | LOC_Os11g35030.1 | 1.5 | 22.719 | Cleavage | cDNA\|growth regulating factor protein, putative, expressed |
| osa-miR396i | LOC_Os11g35030.2 | 1.5 | 22.719 | Cleavage | cDNA\|growth regulating factor protein, putative, expressed |
| osa-miR5801 | LOC_Os11g30020.1 | 0 | 24.131 | Cleavage | cDNA\|retrotransposon protein, putative, Ty1-copia subclass, expressed |
| osa-miR5801 | LOC_Os01g43490.1 | 2 | 21.185 | Cleavage | cDNA\|polygalacturonase, putative, expressed |
| osa-miR164b | LOC_Os06g23650.1 | 1 | 16.58 | Cleavage | cDNA\|no apical meristem protein, putative, expressed |
| osa-miR164b | LOC_Os06g46270.1 | 1 | 20.065 | Cleavage | cDNA\|no apical meristem protein, putative, expressed |
| osa-miR164b | LOC_Os12g41680.1 | 1 | 19.51 | Cleavage | cDNA\|No apical meristem protein, putative, expressed |
| osa-miR1318-5p | LOC_Os03g59790.1 | 0.5 | 24.157 | Cleavage | cDNA\|EF hand family protein, putative, expressed |
| osa-miR1318-5p | LOC_Os03g59770.1 | 0.5 | 21.249 | Cleavage | cDNA\|EF hand family protein, putative, expressed |
| osa-miR1318-5p | LOC_Os04g51610.1 | 1 | 24.158 | Cleavage | cDNA\|calcium-transporting ATPase, plasma membrane-type, putative, expressed |
| osa-miR1318-5p | LOC_Os04g51610.3 | 1 | 24.158 | Cleavage | cDNA\|calcium-transporting ATPase, plasma membrane-type, putative, expressed |
| osa-miR1432 | LOC_Os03g59790.1 | 0.5 | 23.499 | Cleavage | cDNA\|EF hand family protein, putative, expressed |
| osa-miR1432 | LOC_Os03g59770.1 | 0.5 | 20.601 | Cleavage | cDNA\|EF hand family protein, putative, expressed |
| osa-miR397a | LOC_Os01g62490.1 | 0.5 | 20.984 | Cleavage | cDNA\|laccase precursor protein, putative, expressed |
| osa-miR397a | LOC_Os05g38410.1 | 1.5 | 16.443 | Cleavage | cDNA\|laccase precursor protein, putative, expressed |
| osa-miR397a | LOC_Os05g38420.1 | 1.5 | 16.443 | Cleavage | cDNA\|laccase precursor protein, putative, expressed |
| osa-miR397a | LOC_Os11g48060.1 | 1.5 | 15.156 | Cleavage | cDNA\|laccase-22 precursor, putative, expressed |
| osa-miR397a | LOC_Os01g63200.1 | 2 | 19.633 | Cleavage | cDNA\|laccase precursor protein, putative, expressed |
| osa-miR397a | LOC_Os03g16610.1 | 2 | 17.719 | Cleavage | cDNA\|laccase precursor protein, putative, expressed |
| osa-miR397a | LOC_Os07g31310.1 | 2 | 17.895 | Translation | cDNA\|PPR repeat domain containing protein, putative, expressed |
| osa-miR1875 | LOC_Os09g28910.3 | 2 | 13.785 | Cleavage | cDNA\|carbonic anhydrase, chloroplast precursor, putative, expressed |
| osa-miR1875 | LOC_Os09g28910.2 | 2 | 13.785 | Cleavage | cDNA\|carbonic anhydrase, chloroplast precursor, putative, expressed |
| osa-miR1875 | LOC_Os09g28910.1 | 2 | 13.785 | Cleavage | cDNA\|carbonic anhydrase, chloroplast precursor, putative, expressed |
| osa-miR1875 | LOC_Os09g28910.4 | 2 | 13.785 | Cleavage | cDNA\|carbonic anhydrase, chloroplast precursor, putative, expressed |
| osa-miR169k | LOC_Os02g53620.1 | 2 | 17.626 | Cleavage | cDNA\|nuclear transcription factor Y subunit, putative, expressed |
| osa-miR169k | LOC_Os12g42400.2 | 2 | 21.518 | Cleavage | cDNA\|nuclear transcription factor Y subunit, putative, expressed |
| osa-miR169k | LOC_Os12g42400.3 | 2 | 21.518 | Cleavage | cDNA\|nuclear transcription factor Y subunit, putative, expressed |
| osa-miR169k | LOC_Os12g42400.1 | 2 | 21.518 | Cleavage | cDNA\|nuclear transcription factor Y subunit, putative, expressed |
| osa-miR164d | LOC_Os06g23650.1 | 1 | 16.58 | Cleavage | cDNA\|no apical meristem protein, putative, expressed |
| osa-miR164d | LOC_Os06g46270.1 | 1 | 20.065 | Cleavage | cDNA\|no apical meristem protein, putative, expressed |
| osa-miR164d | LOC_Os12g41680.1 | 1 | 19.51 | Cleavage | cDNA\|No apical meristem protein, putative, expressed |
