# Supplementary material for: Alterations in SiRNA and MiRNA Expression Profiles Detected by Deep Sequencing of Transgenic Rice with SiRNA-Mediated Viral Resistance
Source: PLoS One. 2015 Jan 5;10(1):e0116175. doi: 10.1371/journal.pone.0116175 (PMC4283965; doi:10.1371/journal.pone.0116175)
Supplement: S4 Table — Normal count represents the population of miRNA reads without SNP. SNP count presents the population of miRNA reads with SNP (mismatches) from their miRNA precursors. The ratio is calculated by dividing SNP count with normal count. (DOCX) [file pone.0116175.s005.docx]

**Table S4. The SNP ratio of miRNAs among four datasets.** Normal count represents the population of miRNA reads without SNP. SNP count presents the population of miRNA reads with SNP (mismatches) from their miRNA precursors. The ratio is calculated by dividing SNP count with normal count.

|  | **AIA_VF** | **AIA_V** | **T4B1_VF** | **T4B1_V** |
| --- | --- | --- | --- | --- |
| **Normal count** | 7735879 | 6468989 | 4207157 | 4431705 |
| **SNP count** | 418384 | 273183 | 217723 | 214529 |
| **Ratio** | 0.054 | 0.042 | 0.052 | 0.048 |
